# Patterns of non-compliant buprenorphine, levomethadone, and methadone use among opioid dependent persons in treatment

**DOI:** 10.1186/1747-597X-9-19

**Published:** 2014-05-21

**Authors:** Alicia Casati, Daniela Piontek, Tim Pfeiffer-Gerschel

**Affiliations:** 1 Institut für Therapieforschung, Parzivalstr. 25, 80804 Munich, Germany; 2 German Focal Point of the European Monitoring Centre for Drugs and Drug Addiction (EMCDDA), Munich, Germany

**Keywords:** Buprenorphine, Levomethadone, Methadone, Opioid substitution treatment, Non-compliant use, Prescription drugs, Opioid maintenance therapy, Heroin dependence, Opioid dependence

## Abstract

**Background:**

The non-compliant use of opioid substitution treatment (OST) medicines is widespread and well-documented. However, less is known about characteristics of non-compliant OST medicine use and the factors that predict it. The two main goals of this study are to compare characteristics of non-compliant levomethadone, methadone, and buprenorphine use and to explore factors that may differentially predict it among opioid dependent persons in treatment.

**Methods:**

Data from 595 opioid dependent patients with non-compliant OST medicine use were analyzed. Characteristics of use between substances were compared using chi-squared tests and predictive factors were explored through multinomial logistic regressions.

**Results:**

Non-compliant levomethadone and methadone use was characterized by more frequent parallel consumption of other psychoactive substances and intravenous use, whereas buprenorphine was more often procured without a prescription. Regarding predictive factors, methadone was perceived to relieve withdrawal symptoms better than buprenorphine and levomethadone was perceived as being better at modulating the effects of other substances and worst at enhancing mood.

**Conclusions:**

Patterns of non-compliant use differ according to OST medicine. These patterns are considered with the reduction of non-compliant use and the improvement of treatment in mind.

## Background

Methadone maintenance treatment for opioid dependent patients was introduced in Germany in 1988 as part of a pilot study [1]. Since then, opioid maintenance or substitution treatment (OST) has expanded to include buprenorphine in 2000 and diamorphine in 2009 [2] and is considered to be widespread and well-established [1]. It is estimated that the past-year prevalence of problem opioid use in Germany in 2011 was between 0.12-0.34% [3]. The German Federal Institute for Drugs and Medical Devices (BfArM) reports that 75,400 patients underwent OST in 2012, as recorded on census day [4]. This indicates that approximately 30-60% of problem opioid users find themselves in substitution treatment.

In 2011, methadone was the most commonly prescribed OST medicine (54.8%), followed by levomethadone (25.4%) and buprenorphine (19.2%). The remaining less than one percent was made up of dihydrocodeine, codeine, and diamorphine OST prescriptions [5]. Levomethadone and methadone are full opioid agonists while buprenorphine is a partial opioid agonist with a longer half-life. The pharmacological effectiveness of levomethadone and methadone as OST medicines relies on the levorotary form found in both substances. However, their overall effects are not identical as the dextrorotary form in racemic methadone has been found to produce additional side effects [6]. Levomethadone is available in syrup form, methadone in liquid and tablet form, and buprenorphine as sublingual tablets for OST in Germany. OST medicines are mainly delivered by office-based general practitioners in conjunction with community-based pharmacies and a smaller percentage of OST delivery occurs in specialized addiction clinics [7]. There are no differences between the take-home protocols of levomethadone, methadone and buprenorphine regarding regulations for the amount of time, prerequisites or dispensing [8]. However, take-home methadone and levomethadone are dyed and made thicker before being handed out so as to be more difficult to inject [9].

Opioid dependence is characterized by persistent drug-seeking behavior and is often accompanied by poly-drug use and non-compliant OST medicine use. Man et al. [10] found that 66% of opiate users who had overdosed reported mixing opiates with at least one other drug, such as alcohol (14.9%), benzodiazepines (31.1%), or others (8.1%). Specka et al. [11] reported that 90% of opioid users in their study also consumed at least one other psychoactive substance. Cocaine (55%), cannabis (65%), alcohol (60%), and benzodiazepine (53%) use were found to be the most common. Thus, reducing patients’ consumption of psychoactive substances (other than the appropriate consumption of OST medicine) is one of the primary goals of OST [1]. Alongside poly-drug use, OST medicines are often used in ways other than as medically intended. These include intravenous instead of oral or sublingual application, snorting, buying and selling on the black market, and doctor-shopping [12-18]. In order to prevent the latter, BfArM keeps a registry of OST in Germany. In 2012, approximately 160 cases of double opioid substitution treatment (i.e. patient receives two or more OST medicines from different doctors, without doctors’ knowledge) were identified and terminated as soon as the doubling was confirmed [4]. According to the German Medical Association, the five main goals of opioid substitution treatment are to ensure survival, to stabilize patients’ health and treat drug related diseases, to integrate patients in social and work life, to reduce consumption of other addictive substances, and ultimately to free patients from opioid dependence [19].

Although the non-compliant use of OST medicines has been extensively documented [12,13,15-18,20-22], little research has focused on the characteristics of non-compliant OST medicine use and the factors that predict it. A characterization of use and predictive factors could help increase our understanding of the phenomenon and help guide treatment and prevention efforts. Therefore, the two main goals of this study are to compare characteristics of non-compliant levomethadone, methadone, and buprenorphine use and to explore factors that may differentially predict non-compliant OST medicine use among opioid dependent persons.

Regarding the first goal, the following four characteristics of non-compliant use will be compared between OST medicines: 1) parallel consumption of OST medicine and other drugs 2) use of OST medicine not as medically intended 3) intravenous use of OST medicine and 4) procurement of OST medicine without a prescription. Given the likeness in the effects that levomethadone and methadone produce, we hypothesize that the above listed four characteristics of non-compliant use will differ between buprenorphine and levomethadone and buprenorphine and methadone but not between levomethadone and methadone.

Our second goal is to explore predictive factors of non-compliant levomethadone, methadone and buprenorphine use. We will investigate the predictive value of age, gender, frequency of use, and reasons for use. We hypothesize that sedation and anxiety relief will predict non-compliant use of levomethadone and methadone. This hypothesis is based on findings that buprenorphine tends to produce a more “clear head” than methadone, under which patients report a dull feeling and subdued emotions [23,24]. With regards to the predictive effects of age, gender, frequency of use, and other reasons for use, no directed hypotheses can be formulated. The influence of these variables on OST medicine use will be tested in an explorative way.

## Methods

### Data collection

Data from the “Phar-Mon” project collected between January 2007 and August 2011 were used. Phar-Mon is an early warning system that monitors medicine use among patients of outpatient addiction treatment centers. The 34 treatment centers participating in Phar-Mon account for approximately 5% of the outpatient institutions pertaining to the German Addiction Treatment Statistics (DSHS). DSHS is an ongoing monitoring system on substance abuse treatment which includes information on diagnostic data and socio-demographic variables of patients as well as information on the current substance abuse treatment situation in Germany. The sample of centers participating in Phar-Mon was selected randomly. It is a representative sample of the DSHS outpatient centers on the basis of number of patients treated and proportion of opioid dependent patients in treatment. The Phar-Mon project is funded by the German Federal Ministry of Health. For more extensive information on Phar-Mon and its methodology, see references [25,26].

Participant recruitment took place in the outpatient addiction treatment centers. Participation was voluntary, based on informed consent, and not required to receive treatment. Reporting non-compliant OST use did not compromise continuation of treatment as patients were not expected to be compliant upon entry. In accordance with the German Research Foundation (Deutsche Forschungsgemeinschaft - DFG) guidelines [27], ethics approval was not necessary given that the non-experimental design did not expose subjects to risks or high levels of stress. Additionally, subjects were informed of the study’s goals and procedures. Data were recorded and handled anonymously.

The study sample was made up by opioid dependent patients seeking treatment and who were using OST medicines in a non-compliant manner. Opioid dependence was established using ICD-10 criteria for dependence syndrome. Opioid dependent patients were interviewed on their consumption of OST medicines regardless of whether they were already undergoing OST. No further treatment data (e.g. enrollment in OST, first time seeking treatment) were available.

Data were collected by means of the Phar-Mon questionnaire for the following variables: basic demographic variables (i.e. main diagnosis, age, and sex), OST medicine, characteristics of non-compliant OST medicine use (i.e. parallel consumption of OST medicine and other psychoactive drugs which undermine the purpose of OST and jeopardize health; use not as medically intended i.e. other than for the purposes of OST; intravenous use; and procurement without a prescription), consumption frequency, and reasons for use (i.e. to sedate, to induce euphoria, to relieve anxiety, to avoid withdrawal symptoms, to stimulate/arouse, to relieve pain, as a cognitive enhancer, to modulate the effects of other substances, and to enhance/brighten mood). The questionnaire was administered upon treatment entry by trained personnel through face to face interviews. Multiple entries were possible for the characteristics of non-compliant use and for reasons for use. Reasons for use referred specifically to the individual OST medicine consumed, not to reasons for non-compliant use. Data on OST medicine use within the past six months were collected.

The four characteristics of non-compliant OST medicine use (see above) were selected based on the German Federal Medical Association’s guidelines for opioid substitution treatment. These stipulate that patients in OST shall refrain from consuming additional substances that may interfere with the goals of substitution treatment, that OST medicines are to be taken orally/sublingually (with the exception of diamorphine) and not to be handed down or dealt with, and that they are meant to be administered though prescriptions by qualified personnel [19]. Infringements on these regulations, especially when recurrent, may result in discontinuation of OST. Given that patients in this study were beginning treatment, reporting non-compliant OST use did not affect treatment continuation. The criteria for non-compliance were screened on the basis of patients’ self-report. Fulfillment of one or more of the above listed four criteria within the past six months was sufficient to classify the case as non-compliant.

### Statistics

Statistical analyses were performed using SPSS software (version 19). Regarding our first goal (i.e. to compare the characteristics of non-compliant levomethadone, methadone, and buprenorphine use), a chi-squared analysis was performed. Regarding our second goal (i.e. to explore factors that may differentially predict non-compliant OST medicine use), we conducted two multinomial logistic regressions. In the first model, buprenorphine was the reference group and was compared against methadone and levomethadone. For the comparison of methadone against levomethadone, a second model was run with methadone as the reference group. As predictors, age, male gender, frequency of consumption (consumption days within past month), and reasons for use (yes vs. no) were included in the models. Collinearity diagnostics were conducted and evaluated based on the variance inflation factor (VIF) and tolerance. Values of less than 1.4 (VIF) and greater than 0.69 (tolerance) indicated no serious multicollinearity. All statistical tests were performed using a significance level set at .05.

## Results

### Sample

A total of 595 cases of non-compliant OST medicine use was recorded. These cases corresponded to 79 cases of levomethadone use, 297 cases of methadone use, and 219 cases of buprenorphine use (buprenorphine alone = 212 and buprenorphine/naloxone = 7). Descriptive statistics for age, gender, mean days of consumption, and reasons for use for the three OST medicines can be found in Table 1.

**Table 1:** Descriptive statistics for the buprenorphine, levomethadone and methadone groups according to age, gender, consumption frequency, and reasons for use

	**Buprenorphine (n = 219)**	**Levomethadone (n = 79)**	**Methadone (n = 297)**
Age, M (SD)	34.41 (7.92)	37.18 (9.60)	35.13 (8.59)
Gender, % male	90.4	69.6	81.1
Consumption days within past month, M (SD)	23.27 (10.35)	28.58 (6.13)	28.00 (6.13)
Reasons for use, %			
To sedate	50.9	55.7	54.9
To induce euphoria	8.9	5.1	9.1
To relieve anxiety	18.6	21.5	20.6
To avoid withdrawal symptoms	89.4	97.5	97.0
To stimulate/arouse	6.5	5.1	9.9
To relieve pain	5.1	7.6	10.2
As a cognitive enhancer	3.7	5.1	7.2
To modulate the effects of other substances	8.4	24.1	16.4
To enhance/brighten mood	35.0	19.0	30.3

Footnote: The above stated are valid percentages. The maximum number of missings in any group was n = 5.

### Comparison of the characteristics of non-compliant OST medicine use

**Figure 1:**
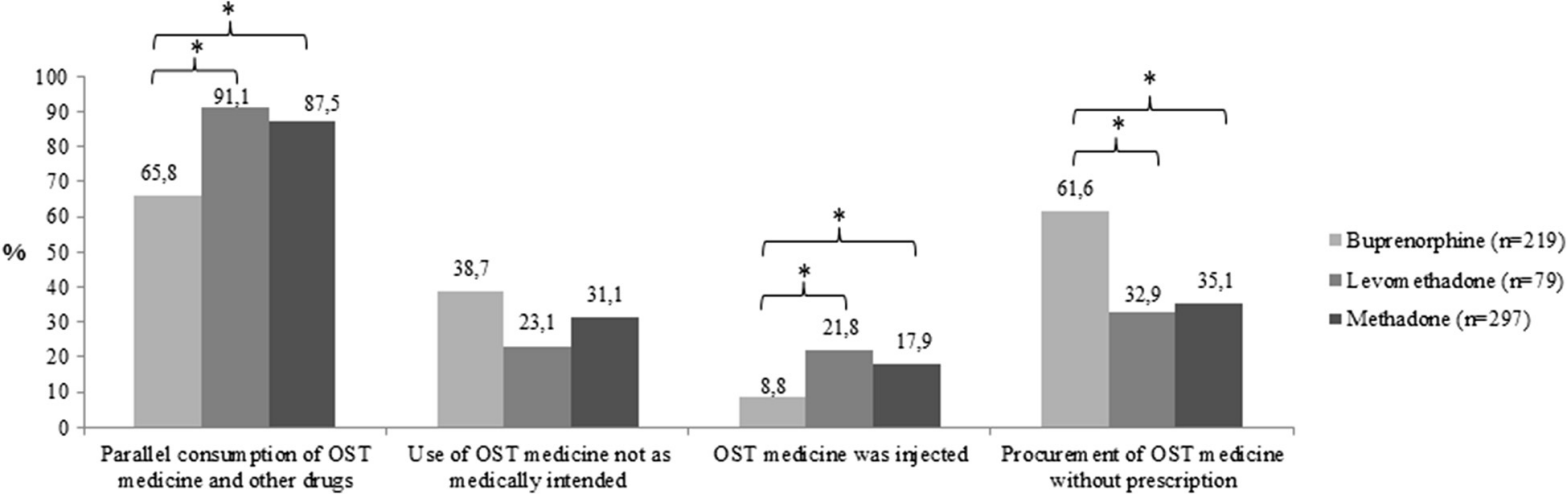
**Characteristics of non-compliant use for buprenorphine, levomethadone, and methadone.** *Symbol denotes p < .05.

Figure 1 provides an overview of the characteristics of non-compliant use for each of the three OST medicines. Parallel consumption of OST medicines and other drugs was significantly more often reported for levomethadone (91.1%) and methadone (87.5%) than for buprenorphine (65.8%) (χ^2^ = 44.47, df = 2, p < 0.001). The individual comparisons for use not as medically intended yielded a marginal difference between buprenorphine (38.7%) and levomethadone (23.1%) that was not significant at the .05 level. Injecting the OST medicine was significantly more common for levomethadone (21.8%) and methadone (17.9%) than for buprenorphine (8.8%) (χ^2^ = 11.25, df = 2, p < 0.005). Finally, buprenorphine (61.6%) was procured significantly more often without a prescription than levomethadone (32.9%) and methadone (35.0%) (χ^2^ = 41.17, df = 2, p < 0.001).

### Exploration of predictive factors for non-compliant OST medicine use

The two multinomial logistic regression models significantly predicted OST medicine use and accounted for 16.9% and 19.6% of the variance, respectively. Table 2 displays odds ratios and confidence intervals for each of the predictor variables with significant results marked with *.

**Table 2:** Factors associated with the non-compliant use of buprenorphine, levomethadone, and methadone, OR (95% CI)

	**Levomethadone vs. buprenorphine**	**Methadone vs. buprenorphine**	**Levomethadone vs. methadone**
Age	1.05 (1.02-1.08)*	1.01 (0.99-1.04)	1.04 (1.01-1.07)*
Male gender	0.21 (0.10-0.43)*	0.45 (0.25-0.80)*	0.47 (0.26-0.85)*
Consumption days within past month	1.07 (1.02-1.13)*	1.06 (1.04-1.09)*	1.01 (0.96-1.06)
Reasons for use			
To sedate	1.46 (0.80-2.68)	1.23 (0.81-1.87)	1.18 (0.68-2.07)
To induce euphoria	0.58 (0.16-2.09)	0.87 (0.42-1.81)	0.67 (0.20-2.23)
To relieve anxiety	0.95 (0.46-1.94)	0.85 (0.52-1.39)	1.11 (0.57-2.16)
To avoid withdrawal symptoms	2.66 (0.57-12.39)	2.84 (1.23-6.58)*	0.94 (0.19-4.58)
To stimulate/arouse	0.84 (0.20-3.46)	1.30 (0.57-3.00)	0.64 (0.17-2.38)
To relieve pain	1.05 (0.34-3.29)	1.88 (0.85-4.14)	0.56 (0.21-1.49)
As a cognitive enhancer	1.93 (0.47-7.97)	1.74 (0.68-4.47)	1.11 (0.31-3.97)
To modulate the effects of other substances	5.73 (2.42-13.55)*	1.86 (0.97-3.60)	3.08 (1.47-6.44)*
To enhance/brighten mood	0.31 (0.14-0.71)*	0.74 (0.45-1.20)	0.42 (0.19-0.92)*

Footnote: Factors that proved to be significant at the .05 level are marked with *. In the first model (first two columns), buprenorphine was the reference group. In the second model (third column), methadone served as the reference group.

### Age and gender

Older age was found to be associated with an increase in the odds of consuming levomethadone over buprenorphine by a factor of 1.05 (95% CI = 1.02-1.08) and levomethadone over methadone by a factor of 1.04 (95% CI = 1.01-1.07). Being male was associated with smaller probabilities of consuming levomethadone than buprenorphine (OR = 0.21, 95% CI = 0.10-0.43), methadone than buprenorphine (OR = 0.45, 95% CI = 0.25-0.80), and levomethadone than methadone (OR = 0.47, 95% CI = 0.26-0.85).

### Consumption frequency

An increase in consumption frequency was associated with an increase in the odds of levomethadone over buprenorphine consumption by a factor of 1.07 (95% CI = 1.02- 1.13) and methadone over buprenorphine consumption by a factor of 1.06 (95% CI = 1.04-1.09).

### Reasons for use

Avoiding withdrawal symptoms was associated with an increase in the odds of consuming methadone instead of buprenorphine (OR = 2.84, 95% CI = 1.23-6.58). Modulating the effects of other substances was associated with an increase in the likelihood of consuming levomethadone over buprenorphine (OR = 5.73, 95% CI = 2.42-13.55) and levomethadone over methadone (OR = 3.08, 95% CI = 1.47-6.44). Finally, the consumption of an OST medicine to enhance/brighten mood was associated with decreased odds of consuming levomethadone than buprenorphine (OR = 0.31, 95% CI = 0.14-0.71) and with decreased odds of consuming levomethadone than methadone (OR = 0.42, 95% CI = 0.19-0.92). Sedating, inducing euphoria, relieving anxiety, stimulating, relieving pain and consuming with the purpose of cognitive enhancement were not found to predict the type of OST medicine used.

## Discussion

More cases of non-compliant methadone use were reported compared to buprenorphine or levomethadone. Although no rates can be calculated from these raw numbers because of the missing total number of patients treated, these findings could suggest a preference for methadone, perhaps due to its larger prescription rates [5]. This could support findings that non-medical prescription drug use is influenced by the availability of a drug [28,29]. However, other factors probably also play a role in misuse, given that the high prescription rates for levomethadone [5] do not correspond to the lower numbers of misuse. Moreover, the abuse potential for buprenorphine/naloxone combination products seems to be lower than for buprenorphine alone [12,30]. Nevertheless, these are just indications and the missing rates do not allow for further interpretations.

Regarding the characteristics of non-compliant OST use, we were able to confirm our hypotheses for the variables parallel consumption, injection, and procurement without a prescription but not for use not as medically intended. We found that levomethadone and methadone are more frequently consumed in conjunction with other psychoactive substances. This parallel consumption may be linked to expectations for modulating the effects of drugs. We also found that levomethadone and methadone were more frequently injected than buprenorphine. In fact, levomethadone syrup was injected almost two and a half times more frequently than buprenorphine sublingual tablets. This is surprising given that take-home methadone and levomethadone syrups are thickened to prevent intravenous (IV) use. While Guichard et al. found that syrup formulations are more difficult to inject whereas buprenorphine in tablet form can be easily injected once crushed and diluted in water [31], our results again suggest the contrary. The precautionary measure implemented for take-home formulations could still reduce IV use, but it does not appear to prevent it. Further research on the influence of medicine form on OST compliance is needed to clarify this question.

Furthermore, our results indicate that in most cases of non-compliant buprenorphine use, the substance was procured without a prescription and that this occurred significantly more often than for levomethadone and methadone. Medicine form could also play a role here, given that buprenorphine in tablet form can be more easily smuggled out of clinics and health care centers to be sold on the black market than the liquid formulations of levomethadone and methadone. However, the availability of each of these OST medicines on the black market is probably also influenced by other factors such as prescription rates and prices. Bell for example found that diversion to the black market occurs in proportion to the amount of OST medicine prescribed to be taken without supervision, and in inverse proportion to the availability of heroin on the market [32].

Regarding the exploration of predictive factors, we found a small effect linking older age to levomethadone use. Differences in the distribution of gender between OST medicines support evidence that males more commonly consume illegal opiates [33]. Males’ tendency for non-compliant use of buprenorphine over methadone and levomethadone could point to gender based preferences for different substances’ effects or differences in prescription patterns for male and female patients.

Our hypothesis regarding the predictive effects of anxiety relief and sedation could not be confirmed. However, the exploratory analysis of reasons for use showed influencing factors that allow for a differential characterization of the three OST medicines. Avoiding withdrawal symptoms was more strongly associated with the consumption of methadone than buprenorphine. In Germany, methadone for OST has been available longer and is prescribed more extensively than buprenorphine. It also dulls and subdues emotions more than buprenorphine [23,24]. This might lead to the perception among opioid dependent patients that methadone is more effective in relieving withdrawal than buprenorphine. To further explore this hypothesis, one could compare non-compliant OST medicine consumption with countries like France, where OST is primarily conducted with buprenorphine [29]. This finding could also point towards too low doses of methadone that result in patients increasing doses as they only experience side effects rather than sufficient withdrawal relief. Unfortunately, we cannot confirm this hypothesis as no mean OST medicine doses were recorded. However, several literature reviews have shown that higher doses of OST medicine are more effective than lower doses in retaining opioid dependent patients in treatment and in reducing concomitant drug and opioid use [34-36]. Current research in Germany found that one-fifth of patients in OST perceived their substitution dosage as being too low [20]. Results of the PREMOS study revealed that one third of patients received OST dosages that were below the minimal maintenance dosage [37].

According to our results, modulating the effects of other substances was more strongly associated with levomethadone than methadone and buprenorphine consumption. We also found concurring evidence that parallel consumption of levomethadone and other substances occurs more often than for methadone and significantly more often than for buprenorphine. This evidence suggests that opioid dependent patients not only believe levomethadone to be better at modulating effects but also consume according to this belief. Levomethadone use was also linked to worst expectations for improving or brightening mood, as compared to the other two OST medicines. To the best of our knowledge, no evidence has been found that the dextrorotary form in racemic methadone could be responsible for opposite effects. Therefore, future studies could further investigate the differential pharmacological effects and side effects of levomethadone and methadone as well as the reputation these substances have among opioid dependent patients according to their perceived effects. The non-compliant use of OST medicines to improve mood suggests that comorbid psychiatric conditions such as depression are not being diagnosed and treated appropriately. The COBRA and PREMOS studies similarly found high rates of psychiatric disorders among opioid dependent persons with proportionally low treatment for these psychiatric disorders [38,39]. This calls for improvements in diagnosing comorbid disorders, especially mood disorders, and in treating patients with antidepressants and psychotherapy when necessary.

In summary, we have found differential patterns of non-compliant OST medicine use. The first main pattern indicates that non-compliant levomethadone and methadone use is characterized by more frequent parallel consumption of other psychoactive substances and intravenous use, whereas buprenorphine is more often procured without a prescription. Second, reasons for use differ between OST medicines in that methadone was perceived to relieve withdrawal symptoms better than buprenorphine, and levomethadone was perceived as being better at modulating the effects of other substances and worst at enhancing mood. Our exploratory analysis yields preliminary results on the factors that predict and characterize non-compliant OST medicine use. However, moderators such as duration of addiction, past treatment, current type and duration of treatment, average OST dose, comorbidity, and psychotropic drug use are still to be explored in order to better understand non-compliance to OST.

The study is limited by certain factors. First, we have no information on the overall number of patients being treated with each OST medicine or on patients’ OST status (e.g. current and past prescriptions, duration of treatment). This makes a calculation of the proportion of non-compliant to compliant users as well as a calculation of rates of non-compliant use for the different OST medicines unrealizable. Additional data would allow for comparisons between groups, enable a country-wide estimate of non-compliance within OST, and clarify the influence of OST status on non-compliant OST medicine use. Second, although patients are assumed to answer honestly because of anonymous, voluntary participation with no repercussions for treatment, we cannot completely discard the possibility of dishonest answers. Third, given the explorative nature of this analysis, we deliberately refrained from adjusting for multiple testing in order to gain as much information as possible on differential patterns of OST use. Future research could build on the results of explorative analyses through experimental research methods (e.g. examining the use of OST medicines by comparing patients in and out of treatment) and by incorporating the above listed additional moderators. Finally, the first two characteristics of non-compliance are relatively open. For the variable “parallel consumption of OST medicine and other drugs”, no information is available on the type of drug used, frequency of use or its detrimental effects. For example, both harmful daily alcohol use and occasional cannabis consumption could have fulfilled this criterion. Further information on drug type could help explain intended effect modulations. Additionally, “use of OST medicine not as medically intended” is a general characteristic that can encompass many different types of use. We relied on the expertise of trained personnel when selecting these categories. The ambiguity of these criteria increases sensitivity but also decreases specificity. Tightening these criteria could yield a more homogeneous group.

## Conclusion

Non-compliance to OST remains a challenge in the treatment of opioid dependent persons. The effects of several moderators on compliance is still to be understood. Our results raise a number of implications for prevention, treatment and policy. First, reducing intravenous consumption of OST medicines and informing consumers of risks related to intravenous use is crucial. Along this note, the continued development of formulations that deter intravenous use and additional investigations into the effects of product form on non-compliant use are necessary to inform changes in the licensing and dispensing of OST medicines. Second, maintenance dosages need to be critically reviewed, especially for methadone. Increasing dosages among patients that are still suffering from withdrawal symptoms is one important way in which clinicians can contribute to patients’ OST compliance. Finally, there are still improvements to be made in diagnosing and treating comorbid psychiatric disorders, especially mood disorders. This would be a major contribution to improving quality of life among opioid dependent persons and could help reduce the non-compliant use of OST medicines as self-treatment of psychiatric symptoms.

## Abbreviations

OST: Opioid substitution treatment; BfArM: Bundesinstitut für Arzneimittel und Medizinprodukte (German Federal Institute for Drugs and Medical Devices); DSHS: Deutsche Suchthilfestatistik (German Addiction Treatment Statistics); VIF: Variance inflation factor.

## Competing interests

This publication will serve as part of AC’s doctoral thesis. DP declares that she received a grant from Lundbeck GmbH for a research project on alcohol epidemiology. TPG has received remuneration for lectures from Essex Pharma GmbH and Molteni-Farmaceutici as well as research funding from Reckitt Benckiser, German federal and state ministries, and the European Commission.

## Authors’ contributions

AC was involved in research conception and design, collection of data, analysis and interpretation of the results, and writing/revision of the manuscript. TP & DP were involved in research conception and design, interpretation of the results, and revision of the manuscript. All authors read and approved the final manuscript.
